# Bird naming systems by Akan people in Ghana follow scientific nomenclature with potentials for conservation monitoring

**DOI:** 10.1186/s13002-015-0062-y

**Published:** 2015-10-31

**Authors:** Justus P. Deikumah, Vida Asieduwaa Konadu, Richard Kwafo

**Affiliations:** Department of Entomology and Wildlife, School of Biological Sciences, College of Agriculture and Natural Sciences, University of Cape Coast, Central Region, Cape Coast, Ghana

**Keywords:** Akan, Conservation potential, Ethnoornithology Ghana, Indigenous knowledge, Scientific nomenclature

## Abstract

**Background:**

Studies on indigenous knowledge of fauna particular birds and its potential use in biodiversity conservation and management are rare globally. Characteristics used in creating indigenous bird names in many Ghanaian languages are undocumented. The main aim of this study is to answer the question “whether indigenous bird naming systems by the Akan tribes in Ghana follow scientific nomenclature and whether indigenous Akan bird knowledge can potentially help improve bird conservation efforts in Ghana.

**Methods:**

Purposive sampling technique was employed in selecting 10 respondents from 25 communities in the five administrative districts in the Central Region. The study was conducted between November 2014 and March 2015. A mixed method approach was adopted in the data collection including key person interviews, focus group discussion, and structured interview supported by a participatory field observation.

**Results:**

Indigenous people in the study area have reported 143 species of birds belonging to 44 families representing 57 % of total number of species with known local names in Ghana. The study revealed that just as Latin and common English naming systems, indigenous Akan bird names originated from features of the bird, including plumage, vocalizations or behavioural characteristics and belief systems of the indigenous people. The study also discovered that indigenous people in the study area have distinct names for different species within a particular family for most of the birds they could identify. However, they occasionally assign a single general name for either the entire family or all species therein.

**Conclusions:**

The study found evidence to support the prediction that indigenous bird naming systems in the Akan language follow scientific nomenclature. Indigenous knowledge and understanding of birds in the study area can be tapped and used in conservation planning and monitoring of birds. This research thus provides sufficient evidence to prove that indigenous knowledge by the Akan tribes in the study area can be useful in bird conservation and monitoring programs in Ghana. Further research in other Ghanaian languages is recommended.

## Introduction

Native and traditional wisdom has historically provided the basis of much of what scientists have documented [1, 2]. Moreover, there is a large body of knowledge about the environment contained in indigenous culture [2, 3]. Indigenous knowledge is based on people’s experience with the environment that are passed from generations to generations usually by word of mouth and cultural ritual [4, 5]. The dynamic nature of indigenous knowledge has assisted communities to survive in their changing environments but its application in biodiversity conservation, monitoring and management has not been fully explored [6].

Anthropogenic environmental change has negatively affected many species including those that are globally threatened [7]. Habitat loss and fragmentation, land use intensification such as agriculture and mining [8, 9], overexploitation of resources population expansion, chemical toxins and pollution, as well as introduced diseases, inter-specific interactions pose major threats to many populations [7]. Biodiversity conservation efforts require an integrated approach with the involvement of local inhabitants as major stakeholders. However, incorporating indigenous knowledge in conservation efforts has been largely neglected in most parts of the world, particularly in the developing world, where threats to many endemic or threatened biodiversity are most tenuous [10].

Most common English names given to birds are derived from certain expressive attributes or behaviours or morphological characteristics. Appearance, vocalization, habitat and food preference, ecological niche and habit of the birds are major origin of most bird names [11]. Just like English names scientific names of birds are mostly descriptions that refer to colour, call, size, shape, flight patterns or number of individuals in flight [12]. For example in Latin *atr*- is the prefix meaning black and *gula* in Latin means throat [13]. Thus the Black chinned sparrow has the scientific name *Spizella atrogularis.* The call of some birds in Latin is used in the scientific naming of these bird species. For example, the Grasshopper Warbler is called *Locustella* because of the supposed resemblance of the birds call and the insects’ chirrup [14].

Similarly vernacular names exist for most species of birds in many parts of the globe. The names are unique and their existence depends on whether or not a bird species is known in a particular locality. Indigenous nomenclature does not follow any particular rule but mostly informed by species’ morphological features and belief systems of the local people [15]. However, the tradition of ascribing names to groups of animals rather than to individual species is prominent in the way indigenous people create bird names.

In Ghana indigenous people use morphological, ecological and behavioural characteristics as well as seasonal occurrence and their association with specific animals to create names for species [15–17]. For example*,* the ‘*Asanteman*’ (people of the Ashanti Kingdom in Ghana) associate themselves with the characteristics of the crested porcupine (*Hystrix cristata*), hence they are called *‘Asante kotoko’* [18]. ‘*Asante’* is the name of the kingdom and ‘*kotoko*’ is the local name of the crested porcupine. They call themselves ‘*kotoko*’ because during ancient wars when one ‘*Asante’* person is killed, thousands of ‘*Asante’s*’ shall come. In literal terms, the defensive mechanism of the porcupine is robust and prevents attack by most major predator [18]. Similarly the ‘*Bretuo*’ clan also in the Ashanti Region of Ghana uses the leopard, (*Panthera pardus),* as their symbol owing the aggressiveness and exceptional bravery of the leopard [18]. The symbol of the ‘*Asona*’ clan of the *‘Okyeman’* tribe in the Eastern Region of Ghana is ‘*kwaakwaadabi*’ the pied crow, (*Corvus albus)* [18]. They believe the crow is characteristically wise and skillful in its foraging strategies as a scavenger. The common kestrel (*Falco tinnunculus),* ‘*sansa’* in the local Akan language is the symbol of the ‘*Oyoko*’ clan in Ghana because they regard the kestrel as patient but confident when searching for food [18]. The animal naming systems in all Ghanaian languages have either not been studied or documented. It is necessary to understand such naming systems particularly for a popular taxon such as birds as we used as case- study species so as to unravel the potentials of indigenous people in bird conservation and monitoring efforts in Ghana.

In this paper, we answered the question “if indigenous bird names follow scientific nomenclature and whether indigenous knowledge of birds can potentially improve conservation efforts in Ghana by examining the bird naming systems by the Akan tribe in Ghana. First, we identified and investigated whether characteristics used by indigenous Akan people in local bird naming systems follow scientific nomenclature. Second, we investigated the potential role indigenous knowledge of Akan communities play in bird conservation efforts in Ghana. To advance ornithology, academic institutions and financial organizations must accept the value of indigenous knowledge to support local initiatives of documenting, assessing and applying indigenous knowledge to biodiversity conservation and development [19, 20].

## Methods

### Study area and tribe

The Akan people belong to a meta-ethnicity and Potou–Tano Kwa ethno-linguistic group in the southern regions of Ghana and the Ivory Coast in West Africa. They are the largest meta-ethnicity and ethno-linguistic group in both countries with a population of > 20,000 000 people. The Akan language (Twi–Fante) is a group of dialects within the Central Tano branch of the Potou–Tano Kwa language family. Subgroups of the Akan people include Asante, Akuapem and Akyem (the Twi dialect), Agona, Kwahu, Wassa, Fante, Assin and Brong.

The study area spans four administrative districts and one metropolitan area. These are Assin North and South districts, Twifo-Hemang lower Denkyira, Komenda-Edina-Eguafo-Abrem and Cape Coast Metropolitan Assembly. Residents in these areas are predominantly fisher folks, farmers and hunters. Their livelihood depended heavily on the forests and biodiversity nearby. Crops cultivated include oil palm, cassava, plantain and other food crops. The local dialect of the residents is predominantly Twi and Fante.

### Study approach and data collection

The study involved field documentation of local names of bird species and literature search of existing documents containing local names of birds that occur in Ghana. The field component of the study was conducted in 25 communities selected randomly from the five districts of dialectic differences. In each community the meaning of bird names, their origin and importance to the Akan were recorded from each of 10 respondents. The field guide of birds of Ghana [21] was relied on for bird images and for confirmation of some of the local names.

Information was gathered from the local people using a guided questionnaire and key person interviews, focus group discussion and personal observation. The following parameters were also investigated; the socio-demography of the respondents (i.e. age, gender, occupation, level of education), the socio-cultural importance of birds and the cultural importance of birds. For easy identification of bird species, respondents were shown picture catalogue of coloured photographs of local birds. Questions were asked verbally in the local dialects with the help of an interpreter where necessary.

### Sampling procedure

A purposive sampling technique was employed in this study with each community visited once between November 2014 and March 2015. A mixed method approach was adopted for data collection including key person interviews, focus group discussion, and structured interview. In each of the 25 communities, 10 respondents were selected purposively summing up a sample size of two hundred and fifty respondents. Information derived from the surveys was used to design a database for further analysis.

### Participatory field observation

Using images of birds alone did not provide enough information about birds to respondents for accurate identification. Due to this limitation, a field-based participatory bird survey approach was designed and conducted using key persons identified to have better local experience in bird identification. This novel approach used in this study enabled local experts to use bird calls, physical appearance, habitat and behaviour of birds within their natural environment as better clues to easily identify some species. The participatory field observation approach improved the accuracy of the data and was useful in discovering inconsistencies in what participants say or often believe is a correct identification of a species.

### Data analysis

Information derived from the field was used to design a database for further analysis in SPSS. Assessment was made on whether all the objectives were achieved mainly by employing descriptive statistics in the analyses. Twenty most common bird species which had a frequency of four or more were selected from the database after running frequencies. Crosstabs were done to assess the twenty most common species listed and the characteristics used in indigenous Akan bird naming system. Crosstabs were also done to assess how varied results are among some parameters in the socio-demography of the respondents. Results were presented in summary tables and graphs. A comparison of the traditional naming system by Akan people with the scientific and English systems helped us to identify the potentials that indigenous naming systems can have for improved of conservation efforts in Ghana. However our comparison does not imply that scientific classification systems are more ‘right’ than the indigenous Akan system.

## Results

Overall 143 species of birds belonging to 44 families were reported and identified correctly by respondents in the study area in local Akan language. The current study revealed that respondents confirmed >51 % of the local names of all 249 species known so far in Ghana in the local Akan language.

Two hundred and fifty respondents were interviewed, out of which 196 were males and the remaining 54 being females. The age of respondents varied between 18 and 78 years but most of them were between 30 and 59 year (>66 %). Greater than 45 % of the local people interviewed had up to secondary level education while 25 and 4.0 % had basic and tertiary level education respectively. The remaining 26 % had no formal education.

A total of 71 % of respondents indicated that they had good knowledge of birds in response to how familiar they were with birds in general and the remaining 29 % had fair knowledge of birds. The survey revealed that each respondent knew at least ten species of birds.

Of the 143 bird species were reported, 8 % were categorized as common to the respondents within all five administrative districts (Table 1). Bird species or species group that was identified by more than four respondents in each community was categorized as common in the study area (Table 1). Of those common species, ‘*nsu-noma’* (referring to species from the following families: Anatidae, Anhingidae, Ardeidae, Charadriidae, Gruidae, Jacanidae, Pelecanidae and Phalacroracidae) had the highest frequency (34) of identification while Dicruridae ‘*kotokosambire’;* Apodidae ‘*ankadaade*’ and Estrildidae ‘*antorowie*’ had the least (4) mentions respectively.

**Table 1:** Frequency of most common bird species groups identified with local names by respondents in different districts

Species group	Local name	Total frequency	Percentage proportion per District				
			AN	AS	THLD	KEEA	CCMA
Weavers	*Akyem*	7	1	2	1	1	2
Kites/falcons	*Akroma*	19	5	8	0	2	4
Crows	*Adene*	12	2	0	1	2	7
Parrots	*Akoo*	7	4	1	0	1	4
Turacos	*Brobe*	7	1	2	3	0	1
Egrets/herons	*Nsu-noma*	34	5	6	7	15	2
Vultures	*Pete*	7	2	1	0	0	4
Egrets	*Belebele*	7	3	0	2	0	2
Bee-eaters	*Bronya-noma*	5	0	0	0	5	0
Doves	*Abuburo*	27	2	14	6	0	5
Hornbills	*Akyenkyena*	10	5	0	1	4	0
Manikins	*Antorowie*	4	0	0	2	0	2
Swallows	*Ankadaade*	4	0	0	3	1	0
Sparrows	*Bola-noma*	7	1	0	1	0	5
Tinkerbirds	*Apiti*	8	2	2	0	3	1
Drongos	*Kotokosambire*	4	1	1	1	1	0
Owls	*Patu*	6	2	0	2	1	1
Nightjars	*Santrofie*	12	0	7	0	3	2
Francolins	*Aboko*	5	1	1	2	0	1
Sunbirds	Aserewa	6	1	0	3	1	1

(AN = Assin North, AS = Assin South, THLD = Twifo-Hemang Lower Denkyira, KEEA = Komenda-Edina-Eguafo-Abriem and

CCMA = Cape Coast Metropolitan Assembly)

However, between districts the identification of birds varied among respondents. All 50 respondents from the KEEA had reported knowledge of the bee-eaters ‘*bronya-noma’* but where species are not recorded in a districts then that species is either not known by the local name or of any characteristics used for the local naming. KEEA recorded the highest number of the egrets/herons (15) while AS recorded the highest number species of doves (14) (Table 1).

### Characteristics used by indigenous Akan tribes in local bird naming

**Fig. 1:**
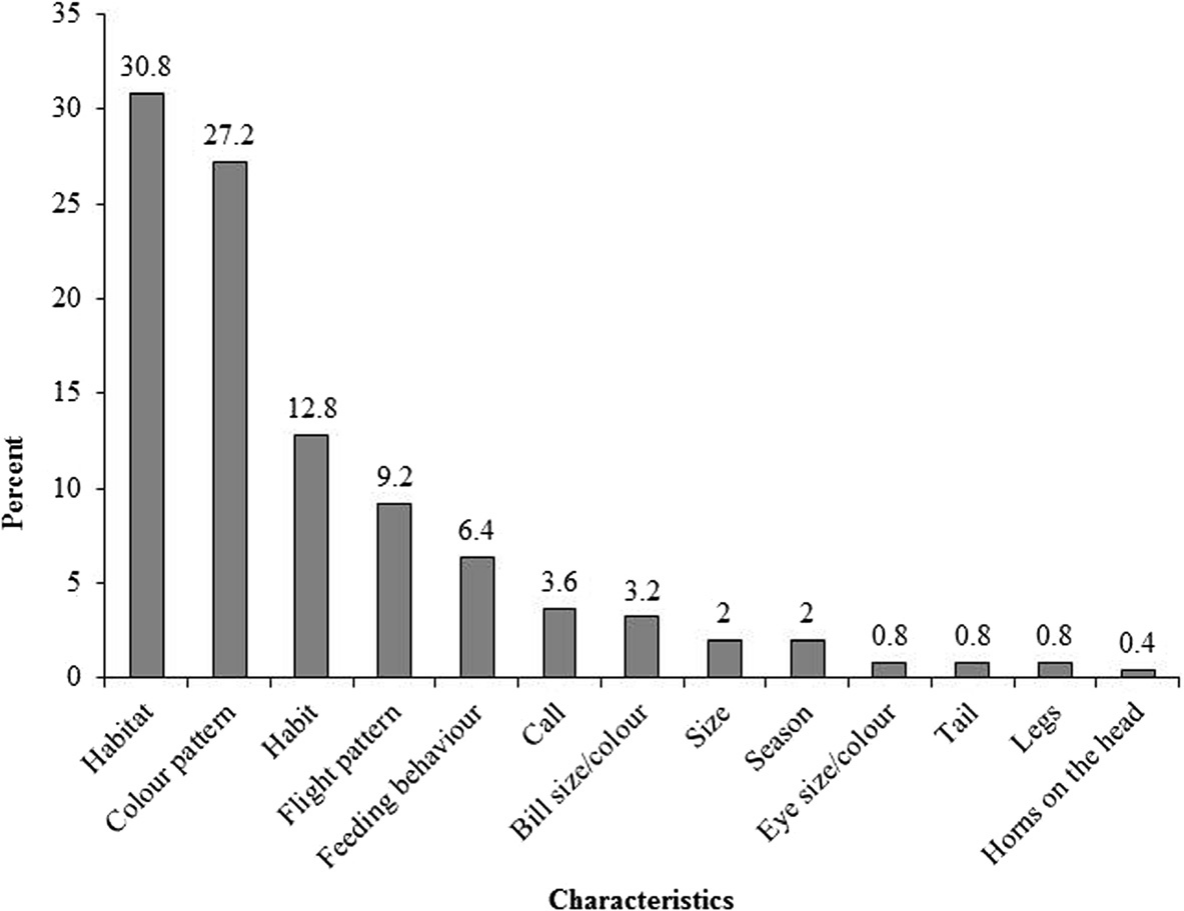
Characteristics used by indigenous Akan people in naming birds

**Fig. 2:**
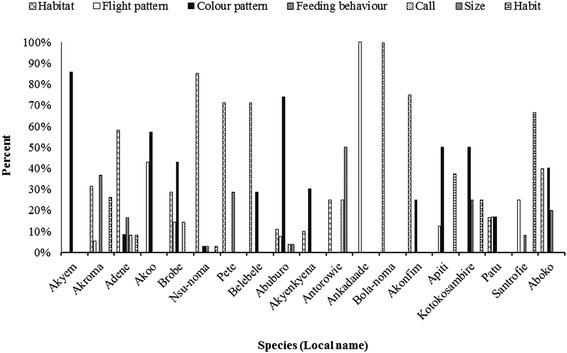
Common characteristics used by indigenous Akan people in naming some common bird species

Greater than 80 % of the locally recognized species have names derived from five major characters including habitat, flight patterns, colour pattern, feeding behaviour and habit or a combination of them (Fig. 1). The most frequent characteristic used was habitat, followed by colour patterns; habit; flight pattern and feeding behaviour. The pied crow, (*Corvus albus)* had three different names namely; ‘*akonkrain’,* ‘*adene’* and *‘kwaakwaadabi’* based on three different characteristics (habit, colour pattern and call) (Fig. 2).

With the exception of ‘*akyem’* (Ploceidae)*,* ‘*bola-anoma’ (*Passeridae) and ‘*ankadaade’* (Apodidae) which were only identified by single characteristics the rest were identified by combinations of different characteristics (Fig. 2). Weavers are generally called ‘*akyem’* and were the only species group which were identified by using only colour pattern (Fig. 2). While swifts and swallows were identified by their flight pattern, the habitat of sparrows was the main characteristic used. The rest of the most common birds were mainly named by combinations of some or all five characteristics (Fig. 2).

### Akan bird naming system could identify different species in different families

**Fig. 3:**
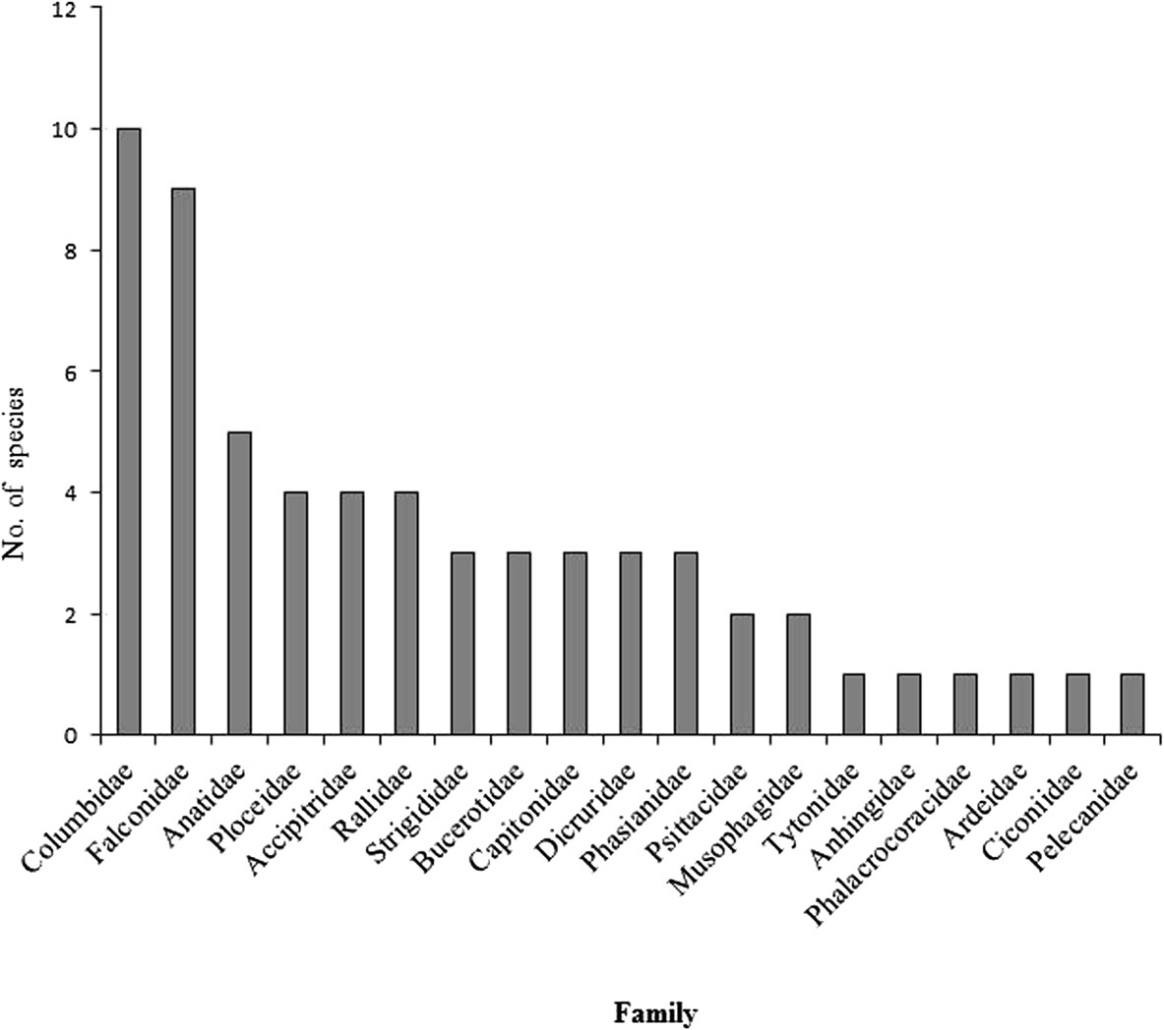
Number of bird species within families reported by respondents

Like Latin and common English bird naming systems, indigenous Akan people have specific names for species within a particular family (Fig. 3 & Table 6). As many as ten species were identified with different local names in the family Columbidae and nine species within the family Falconidae. The following families: Anatidae, Ploceidae, Accipitridae, Rallidae, Strigidae, Bucerotidae, Capitonidae, and Phasianidae had between 4 and 6 species identified by different names based on different characteristics (Fig. 3).

**Fig. 4:**
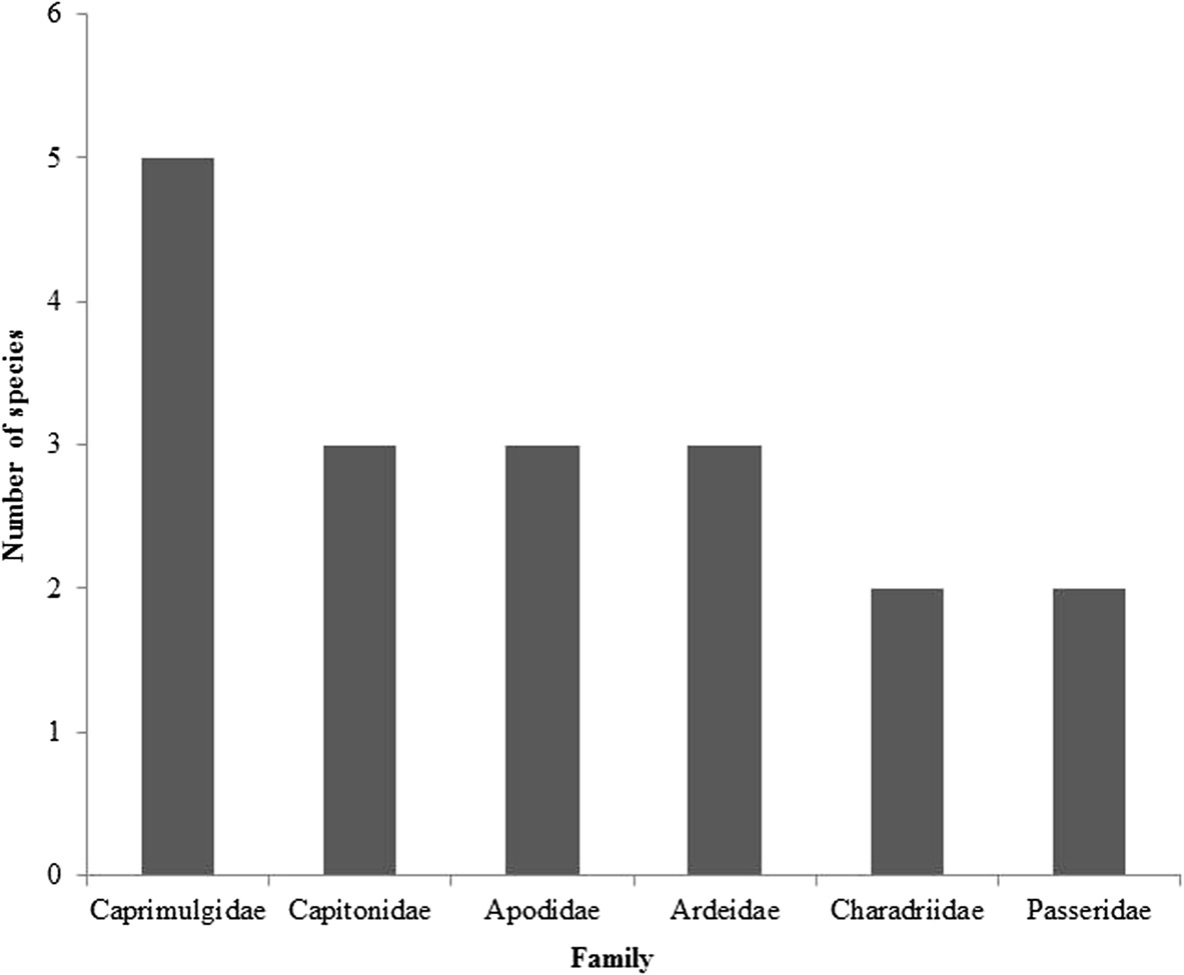
Number of bird species with same local name within families

In contrast, indigenous bird names occasionally used a general term or just one name to refer to either the entire family or genus (Fig. 4). A total of 6 families have all species of birds referred to by just a name. For instance all five species in the family Caprimulgidae (Nightjars) are all identified as ‘*santrofie’* locally while within each of the following families, Ardeidae and Apodidae all 3 species known locally as indicated by respondents have the same names, *‘nsu- noma’* and ‘*ankadaade*’ respectively (Fig. 4). Similarly local people identified two species each in the families Passeridae and Charadriidae but refer to both birds within each family as *‘bola-noma’* and *‘bakamnoma’* respectively (Fig. 4).

### Potential role indigenous knowledge can play in bird conservation

Most respondents have adequate knowledge about the time of day, season of year and the habitat of the species of birds they could identify during this study (Table 2). With the exception of the nightjars and owls which were reported to have been seen in the evening, all other birds were reported to have been seen all the time. Most common birds were seen in both dry and rainy seasons of the year except the bee-eaters which were reported to be seen only in the dry season.

**Table 2:** Bird habitats as reported by indigenous respondents in the study area in percentages

Species group	Local name	Frequency	Around home (%)	Forest interior (%)	Forest edge (%)	Primary forest (%)	Roadside (%)	Cultivation (%)	Around water (%)	Wooded savannah (%)
Weavers	*Akyem*	7	100	71	100	100	100	100	100	100
Kites/falcons	*Akroma*	19	100	79	100	100	100	100	100	100
Crows	*Adene*	12	100	50	100	100	100	100	100	100
Parrots	*Akoo*	7	-	86	29	-	-	-	-	29
Turacos	*Brobe*	7	29	71	86	100	43	71	43	86
Egrets/herons	*Nsu-noma*	34	9	6	9	6	6	12	100	29
Vultures	*Pete*	7	100	57	100	100	100	100	100	100
Egrets	*Belebele*	7	100	43	86	86	100	100	100	100
Bee-eaters	*Bronya-noma*	5	100	40	100	100	100	100	100	100
Doves	*Abuburo*	27	100	74	96	100	100	96	93	96
Hornbills	*Akyenkyena*	10	100	100	100	100	100	100	90	100
Manikins	*Antorowie*	4	75	25	100	100	100	100	100	100
Swallows	*Ankadaade*	4	100	-	100	75	100	100	100	100
Sparrows	*Bola-noma*	7	100	14	86	100	100	100	100	100
Tinkerbirds	*Apiti*	8	88	50	88	100	100	100	100	100
Drongos	*Kotokosambire*	4	25	100	100	100	75	100	100	100
Owls	*Patu*	6	-	100	67	50	-	-	-	33
Nightjars	*Santrofie*	12	83	8	50	58	100	83	58	83
Francolins	*Aboko*	5	20	60	100	100	100	80	40	80
Sunbirds	*Aserewa*	8	88	50	100	100	100	100	63	100

Respondents identified eight habitat types within which they encounter birds in the study area. These include homes, primary forest, forest edge and interior, along roadsides, cultivations, around water bodies and in wooded savannahs (Table 2). Sixty percent of the 20 commonest bird species reported in the study area occur in all eight habitat types. Parrots ‘*akoo*’ occur in only two of the habitat types, namely forest interior and forest edge (Table 2). Swallows ‘*ankadaade*’ occur in all habitats except in forest interior. Owls ‘*patu’* occur in forest interior, forest edge, primary forest and wooded savannah.

Distribution of age groups regarding familiarity of respondents with local bird naming vary among age groups (Table 3). Out of 250 respondents interviewed, the proportion of male to female who showed fair knowledge of local bird names was evenly distributed. Familiarity of birds is inversely proportional to the level of education. Respondents with no formal education (30 %) and those with up to secondary level (38 %) respectively had greater knowledge in local bird naming in the study area.

**Table 3:** Distribution of percentages of familiarity of birds according to age groups

Age group	Frequency	Little (%)	Well (%)	Very well (%)
<20	15	40.0	53.3	6.7
20–29	36	38.9	52.8	8.3
30–39	58	39.7	46.6	13.8
40–49	61	31.1	60.7	8.2
50–59	46	8.7	87.0	4.3
60–69	24	16.7	58.3	25.0
70–79	10	20.0	40.0	40.0
Total	250	28.8	59.6	11.6

Respondents identified some bird species by their common uses and cultural impacts on their communities. Birds like ‘*santrofie*’ and ‘*patuo*’ were reported as indicators of bad omen by > 97 % of all respondents. Almost all respondents agreed that some birds have been used traditionally as symbols and that have positive effect on individuals or the entire community. Out of 250 respondents 79 % said birds are culturally important but the remainder of them disagreed while greater than 96 % accepted that birds are economically important to them (Table 4).

**Table 4:** Importance of birds to indigenous people in the study area

Species	Frequency	Cultural		Religious		Economic	
		No (%)	Yes (%)	No (%)	Yes (%)	No (%)	Yes (%)
*Abuburo/Abronoma/Agyenakuku/Abuburo-somafo*	29	79	21	17	83	0	100
*Adene/Akonkrain/Kwaakwaadabi*	71	0.0	100	99	1	6	94
*Akroma/Akromaboo/Osansa*	8	88	12	100	0	0	100
*Brobe/Kokokyinaka/Aforowa*	9	78	22	100	0	0	100
*Pete/Kokosayi/Kakaaku*	40	0	100	97	3	8	92
*Akokohwede/Aboko*	5	100.0	0	100	0	0	100
*Akonfim*	3	34	66	100	0	0	100
*Santrofie*	5	20	80	100	0	20	80
*Akoo/Awirekwa*	70	2	98	96	4	4	96
*Abonto*	8	88	12	100	0	0	100
*Patuo*	1	0	100	100	0	100	0
*Belebele/Nantwie anoma*	1	100	0	100	0	0	100
Average	-	21	79	88	12	4	96

Respondents identified six threats to birds in the study area (Table 5). All respondents in all occupation groups said habitat destruction was the major threat to birds’ in the study area (Table 5). Farmers, hunters and fishermen declared that hunting birds for food have significantly caused population declines (99.3 %). Less than 36 and 33 % of respondents in all occupation categories respectively, answered ‘No’ to whether road kills and pesticide use were threats to birds in the study area.

**Table 5:** Issues affecting birds as reported by respondents

Occupations	Frequency	Habitat loss	Pollution		Food		Road kills		Pesticides		Building style	
		Yes (%)	No (%)	Yes (%)	No (%)	Yes (%)	No (%)	Yes (%)	No (%)	Yes (%)	No (%)	Yes (%)
Carpenter/Mason/Repairer	18	100	0	100	0	100	27.8	72.2	22.2	77.8	0	100
Unemployed/Students	27	100	11.1	88.9	0	100	29.6	70.4	29.6	70.4	14.8	85.2
Farmers/Hunters/Fishermen	142	100	9.2	90.8	0.7	99.3	31.0	69	26.8	73.2	14.1	85.9
Teacher/Health assistants	35	100	20	80	0	100	22.9	77.1	31.4	68.6	8.6	91.4
Driver	19	100	15.8	84.2	0	100	36.8	63.2	21.1	78.9	15.8	84.2
Farmer/Trader	9	100	0	100	0	100	11.1	88.9	0	100	0	100

## Discussion

Bird names are mostly derived from certain expressive attributes or morphological characteristics [22]. This current study shows that Akan local bird names originated from some features of the bird, including plumage, vocalizations, behavioural traits as well as certain belief systems of the various groups in the study area. The study revealed that indigenous people in the study area have distinct names for different species within a particular family for most of the birds they could identify. However, they occasionally assign a single general name for either the entire family or all species therein.

The study also provide sufficient evidence to support the prediction that indigenous bird naming systems in the Akan language follow some sort of scientific nomenclature just as the common English and Latin names have. The study also shows that locals have adequate knowledge of birds within the area. Akan communities show knowledge of birds in general including places the birds occur, particular time the birds are seen and their food preferences. The study also discovered that local people are well informed about current environmental threats affecting birds but may have no concern about how their daily activities affect the birds. This research thus provides sufficient evidence to prove that indigenous knowledge by the Akan tribes in the study area can be useful in bird conservation and monitoring programs.

### Characteristics used by indigenous Akan tribes in local bird naming

Local bird naming by indigenous Akan tribes were mostly descriptions that refer to morphological, geographic, environmental and behavioural characteristics. Morphologically, indigenous Akan tribes used colour pattern, bill size/colour, eye size/colour, tail/colour, legs/size and head/horns to name most birds. For instance all malimbes are called ‘*akyem-polis’* by the respondents in the study area because of its red and black coloration. *‘Polis’* literally means police and they refer to the old Ghana police uniform in which they wore a red hat.

Geographical distribution, habit, climatic as well as other environmental traits was also used to create bird names. For instance, the northern grey-headed sparrow is called ‘*bola-noma’* because it occurs around refuse damps. Hence the name ‘*bola’* which means refuse damp and ‘a*noma’* means bird. All bee-eaters are called ‘*bronya*-*noma’* because of the season they find it around them. ‘*Bronya’* means Christmas in the local Akan language and they refer to these birds as such because they only see them in southern part of Ghana during the dry season. This corroborates the scientific classification of bee-eaters as seasonal migrants [23].

Attributes of birds such as flight pattern and feeding behavior was important in local bird names. For instance the common bulbul is called ‘*apetupre’*, literally meaning to fly abruptly. All birds of preys including kites, falcons and kestrels are referred to as ‘*akroma’* because they hunt and feed on other smaller vertebrates. Other bird names are derived from local understanding of the bird calls e.g. ‘*kwaakwaadabi’,* derived from the sharp ‘*kwaar-kwaar’* sound of the pied crow*,* ‘*sikapeyeya’* derived from farmers interpretation of the call of Klass’s cuckoo which means “money is hard to come by” and the ‘*dweedwee’* call made by the red-bellied paradise flycatcher is its local name.

All sunbirds are called ‘*aserewa’* which literally means small size but to distinguish the different species of the sunbirds they used adjectives describing certain morphological features. They distinguish sunbirds into ‘*aserewa-ogyadawobo’,* ‘*aserewa-sika’,* ‘*aserewa-sikansuo’.* All names of the sunbirds have used both size and colour combinations to give the species name. ‘*Ogyadawobo’*, ‘*sika’*and ‘*sikansuo’*are names referring to colour pattern of the sunbirds, where ‘*ogyadawobo’* means fire on the chest referring to the scarlet-chested sunbird, ‘*sika’* means gold referring to the male superb sunbird and ‘*sikansuo’* literally means the colour of the surface of water in mining areas as a result of the minerals in the soil and that is referring to the female splendid sunbird. This finding suggested that local bird naming systems are similar in composition and structure to the common English names.

### Akan informants could identify different species in different families

Akan informants could identify different species of birds within the same family. For instance all doves in the family Columbidae are called ‘*abuburo’* or ‘*abronoma’* but they can distinguish between the different species within this family with certain adjectives that describes certain morphological of doves. They use either ‘*fi’* which means dirt, ‘*kwao’* is a Ghanaian name for a male or ‘*somafo’* meaning a messenger. They called the Laughing dove ‘*abuburo-fi’* which literally means dirty dove because of the plumage and Tambourine dove ‘*abuburo-tawiah’* (where ‘*tawiah’* means a younger one after a twin, because of the small size of the bird). The Black-billed wood dove is called *‘abuburo-kwao’* because they believe they are the males of the doves and Namaqua dove ‘*abuburo-somafo’* because they claim they were used as messengers in the olden days. The Red-eyed dove is the only species under the family Columbidae which is named by its call ‘*agyenkuku’*.

All birds from the families Anatidae, Anhingidae, Ardeidae, Charadriidae, Gruidae, Jacanidae, Pelecanidae and Phalacroracidae are called by the same name as ‘*nsu-noma’* (meaning water bird). These families are lumped together probably because the local people in the study area are not familiar with these species as most of them do not occur there. This is not surprising because most species in the above mentioned families are restricted to water to some extent or are known to be partially migratory [21, 23]. Moreover, people’s knowledge about water birds or forest birds is dependent on how closer the person is to a water body or a forest.

This study also found that some groups of bird species in the same family have been given the same name. For instance all members of the family Caprimulgidae are called ‘*santrofie’.* Identification and classification of organisms with local names can be problematic when comparing different species [24]. This is a major limitation in Akan indigenous naming system probably due to the fact that local names of birds can create some sort of confusion when most of the names are derived from colour of the plumage or parts of the bird. Perception of colour can be highly subjective and dependent on individuals as what someone may refer to as brown others could perceive it as a different colour.

### Bird naming system by indigenous Akan people conform to scientific nomenclature

The results of this study revealed that bird naming systems by indigenous Akan tribes in Ghana conform to scientific nomenclature. For instance the pied crow from the family Corvidae has three different names among different communities, thus ‘*adene’,* ‘*akonkrain’* and ‘*kwaakwaadabi’*. Each of this names used different characteristics to name the crow. This is similar to the Latin and common English naming of the crow. *Corvus albus* is the Latin name for the pied crow. In Latin ‘*Corvus*’ means a scavenger and ‘*albus’* means white [25]. The Latin name of the crow was derived from both the feeding behaviour and the colour pattern just like the common English name where the ‘pied’ in the name of the crow refers to the black and white plumage of the bird. However, a comparison of the scientific nomenclature to indigenous names of the crows revealed that it followed some sort of scientific approach. The different names were as a result of dialectic differences among communities but these names may have originated from different characteristics. ‘*Akonkrain’* as a scavenger; ‘*adene,’* the black and white colouration while ‘*kwaakwaadabi*’ from the call.

In few cases indigenous Akan names distinctively name only those species they interact with but group the rest together as one species and call them by the same name as in the case of barbets in the Family Capitonidae. The Red-rumped tinkerbird is called ‘*apiti-donko’* and the Yellow-spotted tinkerbird; ‘*apiti asafohene’*; but all other tinkerbirds in this family including Yellow-throated tinkerbird, and speckled tinkerbird are commonly identified and named as ‘*apiti*’ (see Table 6). This contradicts the common English and Latin naming systems where each known species and subspecies has specific epithets that distinguishes them from all other species [26]. This is a major drawback in the local naming system as discovered in this present study and requires further investigation.

**Table 6:** List of birds’ Family name, Common English name and Local name

Family	English Name	Akan name	Derivative
Ploceidae	Red-headed Weaver	*Akyem polis*	Weaver that have red colour on the head as the olden Ghana police
	Little Weaver	*Akyem*	-
	Zitting Cisticola	*Akyem-sebo*	Weaver which have colour pattern similar to the tiger
	Maxwell's Black Weaver	*Akyem-sibere*	Weaver with all black colour
Accipitridae	Hooded Vulture	*Pete*	Ugly
	Hooded Vulture	*Kaakaku*	Ugly
	Black Kite	*Sansankroma*	Hunts small birds
	Short-toed Snake Eagle	*Akromakyekyewa*	Bird of prey
Falconidae	Yellow-billed Kite	*Osansa*	Bird of prey
	Black Kite	*Sansankroma*	Hunts small birds
	Grey Kestrel	*Osansa*	Bird of prey
	African Hobby	*Akromaboo*	Strongest of the falcon
	Red-footed Falcon	*Osansa*	Bird of prey
	Bat Hawk	*Akromaboo*	Strongest of the falcons
	Yellow-billed Kite	*Akroma*	Bird of prey
	Fox Kestrel	*Osansa*	Bird of prey
	Lanner Falcon	*Akromaboo*	Strongest of the falcon
Strigididae	Shelley's Eagle Owl	*Patu-busufo*	Owl of bad omen
	Fraser's Eagle Owl	*Patu-waben*	Owl with horns
	Sandy Scops Owl	*Patu*	Owl
Tytonidae	Barn owl	*Patu*	Owl
Corvidae	Pied Crow	*Akonkrain*	-
	Pied Crow	*Adene*	-
	Pied Crow	*Kwaakwaadabi*	Daily call of the crow
Psittacidae	Grey Parrot	*Akoo-papa*	The real parrot
	Red-fronted Parrot	*Awirekwa*	-
	Red-fronted Parrot	*Awirekwa*	-
Musophagidae	Great Blue Turaco	*Kokokyinaka*	Bird call
	Yellow-billed Turaco	*Aforowa/Brobe*	-
Anatidae	Egyptian Goose	*Nsu-dokodoko*	water goose
Anatidae	Common Shelduck	*Nsu-dokodoko*	Water goose
Anatidae	Common Teal	*Epo-noma*	Sea bird
	White-faced Whistling Duck	*Nsu-dokodoko*	Water goose
	Egyptian Goose	*Nsu-dokodoko*	Water goose
Anhingidae	African Darter	*Nsu-noma*	Water bird
Phalacrocoracidae	Long-tailed Cormorant	*Sukonkon*	Surface of water
Ardeidae	Cattle Egret	*Nantwie-noma*	Mutualistic association of the bird and the cattle
	Green-backed Heron	*Nantwie-noma*	Mutualistic association of the bird and the cattle
	Black Heron	*Nantwie-noma*	Mutualistic association of the bird and the cattle
	Goliath Heron	*Sohori*	Legs of giraffe
Ciconiidae	Marabou Stork	*Nsu-pete*	Water vulture
Rallidae	Grey-throated Rail	*Nsu-noma/Okoto-pue*	Water bird/crab come out
	White-spotted Flufftail	*0Koto-pue*	crab come out
	Buff-spotted Flufftail	*Nsu-noma*	Water bird
	Nkulengu Rail	*Kwakudonsuro*	Love does not fear
Charadriidae	African Wattled Lapwing	*Bakamnoma*	sea bird
	White-headed Lapwing	*Bakamnoma*	Sea bird
Apodidae	Mottled Spinetail	*Ankadaade*	Never lands
	Cassin's Spinetail	*Ankadaade*	Never lands
	African Palm Swift	*Ankadaade*	Never lands
Pelecanidae	Great White Pelican	*Nsu-noma/Okoto-pue*	Water bird/crab come out
Caprimulgidae	Black-shouldered Nightjar	*Santhrofie*	Trouble should happen at home
	Plain Nightjar	*Santhrofie*	Trouble should happen at home
	Freckled Nightjar	*Santhrofe*	Trouble should happen at home
	Brown Nightjar	*Santrofie*	Trouble should happen at home
	Red-necked Nightjar	*Santhrofie*	Trouble should happen at home
Passeridae	Northern Grey-headed Sparrow	*Bola-noma/Akasanoma*	Bird at damp site/ talking bird
	Bush Petronia	*Bola-noma*	Bird at damp site
Bucerotidae	White-crested Hornbill	*Asokwaa*	*-*
	White-crested Hornbill	*Kokoase akokora*	Old man in the cocoa farm
Capitonidae	African Pied Hornbill	*Akyekyena*	
	Red-rumped Tinkerbird	*Apiti donko*	Slave of the tinkerbird
	Yellow-throated Tinkerbird	*Apiti*	*-*
	Yellow-rumped Tinkerbird	*Apiti*	*-*
	Speckled Tinkerbird	*Apiti*	*-*
Nectariniidae	Yellow-spotted Tinkerbird	*Apiti asafohene*	Royal majesty of the tinkerbird
	Scarlet-chested sunbird	*Aserewa-ogyadawobo*	Sunbird with fire on the chest
		*Aserewa-sika*	Sunbird with gold belly
		Aserewa-sikansuo	Sunbird with multi-colours like water with minerals
Dicruridae	Fork-tailed Drongo	*Kotokosambire*	
	Shining Drongo	*Mekanmese-kwakye*	
Phasianidae	Stone Partridge	*Aboko*	-
	Ahanta Francolin	*Akokohwede*	Sweet meat
	Latham's Forest Francolin	*Aboko*	*-*
	White-breasted Guineafowl	*Kwaem akokoninpon*	Forested guinea fowl
Columbidae	Bruce's Green Pigeon	*Aboranoma*	*-*
	Tambourine Dove	*Abuburo-tawiah*	Younger dove
	Blue-spotted Wood Dove	*Abuburo-kwao*	Messenger dove
	Namaqua Dove	*Abuburo-somafoo*	Messenger dove
	Black-billed Wood Dove	*Abuburo-kwao*	Male dove
	Laughing Dove	*Abuburo-fi*	Dirty dove
	Blue-spotted Wood Dove	*Abuburo*	-
	Western Bronze-naped Pigeon	*Abuburo*	-
	Red-eyed Dove	*Agyenkuku*	Call of the bird

### Cultural and religious association of the Akan people with birds

Owing to cultural and religious associations, some bird species were considered sacred and may not be killed, eaten or touched. They are regarded as symbols of an existing intimate and/or unseen relationship of a particular clan. The vulture ‘*pete’,* is the totem of the ‘*Asakyir’* clan in Ghana. In some communities, the vulture is accorded no respect and it is being ridicule as lazy, ugly and filthy. During this study people from the communities claim that the vulture is of no important to them so such bird species should be wiped off. This common notion may probably have contributed to the rapid decline in vulture populations leading to their current IUCN listing of critically endangered (CR) [27]. Under such circumstances it is imperative to highlight the positive aspect of the vulture so as to promote efforts to conserve them [28]. Other bird species including crows and nightjars are used in traditional medicine. For instance, eye of crow used as an antidote to cure spiritual ailment and the tail feathers of nightjars used to reverse curse on a person and the faeces is used to cure madness. Such exploitation of species may threaten their survival and may lead to population declines since demand for birds and their parts for these purposes may increase with human expansion. Conservation ecologist have an added responsibility of changing peoples’ attitude towards birds with traditional hatred by educating people about the ecosystem services they provide such as cleaning up carcasses in the environment [15].

### Conservation implications

Indigenous Akan tribes in the study area have shown evidence that their knowledge of bird naming can be tapped for conservation purposes. This study found that birds are important socio-economic and cultural resources to the Akan peoples. The study also provides a preliminary understanding of how local Akan communities perceive, use and traditionally conserve birds in their own ways. This current study supported the earlier classification of the importance of birds to local people in the study area in to the following three categories: consumptive use, non –consumptive use and socio-cultural uses. Community members who frequently interact with or utilize birds are more knowledgeable about the birds. On the basis that indigenous Akan tribes know the location, time and seasonal of occurrence of most birds as well as threats to birds including a fair knowledge about local population declines it is crucial to tap into such indigenous understanding of nature in conservation planning and monitoring of birds in the study area. However, utilizing this information for biodiversity conservation should be done with some caution related to individual bias in knowledge.

## Conclusion

Ethical conservation through indigenous knowledge has foundations in the value systems of most religions and philosophies and is readily understood by the general public [29]. Therefore, local people who support conservation in their own perspective can be inspired to take the lead in protecting biodiversity. The study improved our recognition of the value of indigenous bird knowledge as a tool that can fill in some of the yawning gaps in our knowledge of birds and provide a useful tool for bird conservation. Improved conservation efforts are related to the cooperation among scientists with indigenous people whose livelihood are solely linked to the fates of avifauna [30]. It is recommended that this study is extended to other Ghanaian languages and also to other faunal taxonomic groups to provide detailed understanding of how local naming systems of animals in Ghana can be used in biodiversity conservation. This would be a step in the right direction towards involving local people in conservation of biodiversity in Ghana and Africa.
